# A Systematic Framework for Drug Repositioning from Integrated Omics and Drug Phenotype Profiles Using Pathway-Drug Network

**DOI:** 10.1155/2016/7147039

**Published:** 2016-12-26

**Authors:** Erkhembayar Jadamba, Miyoung Shin

**Affiliations:** ^1^Bio-Intelligence & Data Mining Laboratory, Graduate School of Electrical Engineering and Computer Science, Kyungpook National University, 1370 Sangyeok-dong, Buk-gu, Daegu 702-701, Republic of Korea; ^2^School of Electronics Engineering, Kyungpook National University, 1370 Sangyeok-dong, Buk-gu, Daegu 702-701, Republic of Korea

## Abstract

Drug repositioning offers new clinical indications for old drugs. Recently, many computational approaches have been developed to repurpose marketed drugs in human diseases by mining various of biological data including disease expression profiles, pathways, drug phenotype expression profiles, and chemical structure data. However, despite encouraging results, a comprehensive and efficient computational drug repositioning approach is needed that includes the high-level integration of available resources. In this study, we propose a systematic framework employing experimental genomic knowledge and pharmaceutical knowledge to reposition drugs for a specific disease. Specifically, we first obtain experimental genomic knowledge from disease gene expression profiles and pharmaceutical knowledge from drug phenotype expression profiles and construct a pathway-drug network representing a priori known associations between drugs and pathways. To discover promising candidates for drug repositioning, we initialize node labels for the pathway-drug network using identified disease pathways and known drugs associated with the phenotype of interest and perform network propagation in a semisupervised manner. To evaluate our method, we conducted some experiments to reposition 1309 drugs based on four different breast cancer datasets and verified the results of promising candidate drugs for breast cancer by a two-step validation procedure. Consequently, our experimental results showed that the proposed framework is quite useful approach to discover promising candidates for breast cancer treatment.

## 1. Introduction

Developing and discovering a new drug is a very costly and time consuming process, which can take 10–17 years with a cost of 1.3 billion dollars. Despite large investments in research and development each year, there are still only a small number of new drugs approved successfully by the Food and Drug Administration (FDA) each year. Increasing failure rates, high costs, and the lengthy testing process for drug development have led to a process called drug repositioning [1], which refers to identifying and developing new uses for existing drugs to reduce the risk and cost.

Traditional drug repositioning methods primarily use information on chemical structure, side effects, and drug phenotypes and explore similar drugs based on the assumption that structurally similar drugs tend to share common indications [2–4]. In other words, the key idea behind these approaches is that molecularly similar drug structures often affect proteins and biological systems in similar ways [4]. For example, Swamidass [5] used chemical structure data to identify unexpected connections between a known drug and a disease and explored the hypothesis that if a drug has the same target as a known drug, then this new drug would also have activity against the disease. As another approach, Keiser et al. used 3665 US FDA-approved and investigational drugs that together had hundreds of targets, defining each target by its ligands. The chemical similarities between the drugs and ligand sets predicted thousands of unanticipated associations, which have been used to develop new indications for many drugs.

Alternatively, some approaches use a drug phenotype, which is the expression profile of patients undergoing treatment with a drug. For example, the Connectivity Map (CMap) [6, 7] project is exploring the effects of a large number of FDA-approved chemicals (1309 drugs) on gene expression, and these effects are measured in four different cell lines, allowing researchers to analyze the different expression patterns of drug's target genes. Many computational approaches have been introduced to reposition drugs using CMap by analyzing drug-associated expression signatures to match a repositioned drug's effect with a shared perturbed gene expression profile for another disease, under the assumption that drugs that share similar CMap expression signatures have similar therapeutic applications. Using the CMap data, Iorio et al. [8] developed a drug repositioning method by constructing a drug-drug similarity network using gene set enrichment analysis (GSEA) [9] that could compute the similarity between pairs of drugs. Several different studies [3, 10–13] showed that using CMap expression profiles with a combination of various data sources such as drug target databases, drug chemical structures, and drug side effects was an improvement over the current drug target identification methods.

Moreover, the rapid developments in genomics and high-throughput technologies have produced a large volume of disease gene expression profiles, protein-protein interactions, and pathways. The high-level integration of these resources using network-based approaches is reported to have great potential for discovering novel drug indications for existing drugs [14]. For example, Chen et al. [15] introduced two different inference methods for predicting drug-disease associations based on basic network topology using a bipartite graph constructed from DrugBank [16] and Online Mendelian Inheritance in Man (OMIM) [17]. Emig et al. [18] integrated gene expression profiles, drug targets, disease information, and interactions for drug repositioning. Hu and Agarwal [19] created a disease-drug network using disease microarray datasets and predicted new indications for existing drugs using their disease-drug network.

Although many of the above methods have shown encouraging results for finding new indications for old drugs, there are still some limitations. For example, Yildirim et al. [20] concluded that most drugs with distinct chemical structures target the same proteins, and Keiser et al. [21] reported that structurally similar drugs may also target proteins with dissimilar functions, stating that using chemical structure alone is insufficient for successful drug repositioning [22]. In addition, care should be taken when using only the drug phenotype (drug treated) expression profile (such as CMAP) for drug repositioning because some portion of the genes or pathways that show statistically significant expression differences in cell lines treated with the drug may be expressed only because of the drug's side effects or toxicity. Furthermore, the genes expressed in the drug treated profiles for specific disease cell line or tissue only represent a small subset of the biological pathways, whereas the cooperation of genes plays an important role in complex diseases such as cancer. Pathway-based drug repositioning may be a better alternative for drug repositioning for specific diseases such as cancer.

To overcome the above limitations, the current drug repositioning methods require a comprehensive and efficient computational drug repositioning approach that incorporates powerful machine learning approaches using the high-level integration of available data such as disease gene expression profiles (disease profile), drug treated expression profiles (drug phenotype profile), and drug databases (STITCH [23], DrugBank [16], therapeutic target database (TTD) [24]) to discover new drugs for a human diseases. In this study, we aim to develop a systematic computation framework that repositions drugs by employing disease profile and drug phenotype profiles on the drug network along with integrated omics data.

## 2. Materials and Methods

**Figure 1:**
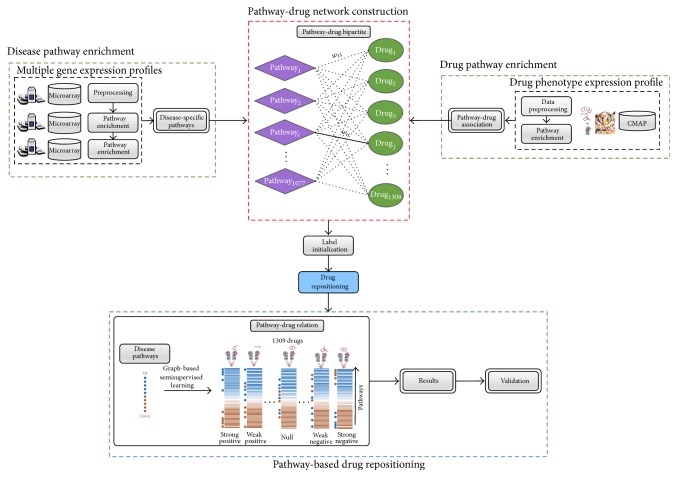
The proposed framework for drug repositioning. The proposed framework consists of several steps. First, disease-specific pathways are identified by disease pathway enrichment of multiple expression profiles for the disease of interest. Second, the drug pathway network is constructed from the drug pathway associations obtained from the drug phenotype profiles. Once the network is constructed, initial labels are assigned using disease-specific pathways and known drugs associated with the given disease. Finally, pathway-based drug repositioning is performed using semisupervised network propagation. The identified drugs are evaluated, and the final results are obtained.

In the framework as shown in Figure 1, we firstly identify* disease-specific pathways* by using an integrative analysis of multiple disease gene expression profiles and construct a* pathway-drug network* structure using pathway-drug associations derived from the CMap* drug phenotype profile*. Then to discover promising candidates, for drug repositioning, we initialize node labels for the pathway-drug network using identified disease pathways and known drugs associated with breast cancer and perform network propagation in a semisupervised manner.

In the following, the detailed explanations of our proposed framework for repositioning and evaluation method are described.

### 2.1. Finding Disease-Specific Pathways from Multiple Disease Expression Profiles

To identify disease pathways related to a specific disease, conventional approaches have usually focused on identifying enriched pathways between cases and controls using data from a single experiment. Specifically, when using real experimental data such as microarray gene expression data, it is possible for different studies to report different results for disease-specific pathways. That is, the results are often not reproducible or not robust even to the mildest data perturbation, so the integrated analysis of multiple existing studies can increase the reliability and generalizability of results [25]. To address these issues, our approach identifies a disease-specific pathway based on disease pathway enrichment using multiple gene expression profiles for a given phenotype, in which the disease pathway enrichment results are integrated. Each disease expression profile is preprocessed, and the pathways that show significant differences between case and control samples are identified by GSEA [9], which returns the enrichment score (ES) and nominal *p* value for each pathway. These scores are used for comparison analysis across pathways to detect significant pathways.

**Figure 2:**
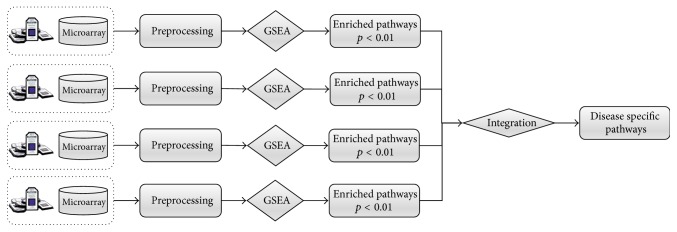
Disease pathway enrichment. Disease-specific pathways are identified from multiple gene expression profiles for the same disease. For each profile, enriched pathways with *p* < 0.01 are selected and integrated by taking their union. The resulting pathways are considered disease-specific pathways for the given disease.

Here, we considered that the integration of pathways significantly enriched for each expression profile could better represent “disease-specific pathways” for the phenotype of interest. To integrate, the pathways with a nominal *p* value less than 0.01 (*p* < 0.01) are selected as significant pathways for each expression profile, and their union is defined as “disease-specific pathways.” Figure 2 presents an illustration of the integration process.

### 2.2. Deriving Pathway-Drug Associations from CMap Drug Phenotype Profiles

**Figure 3:**
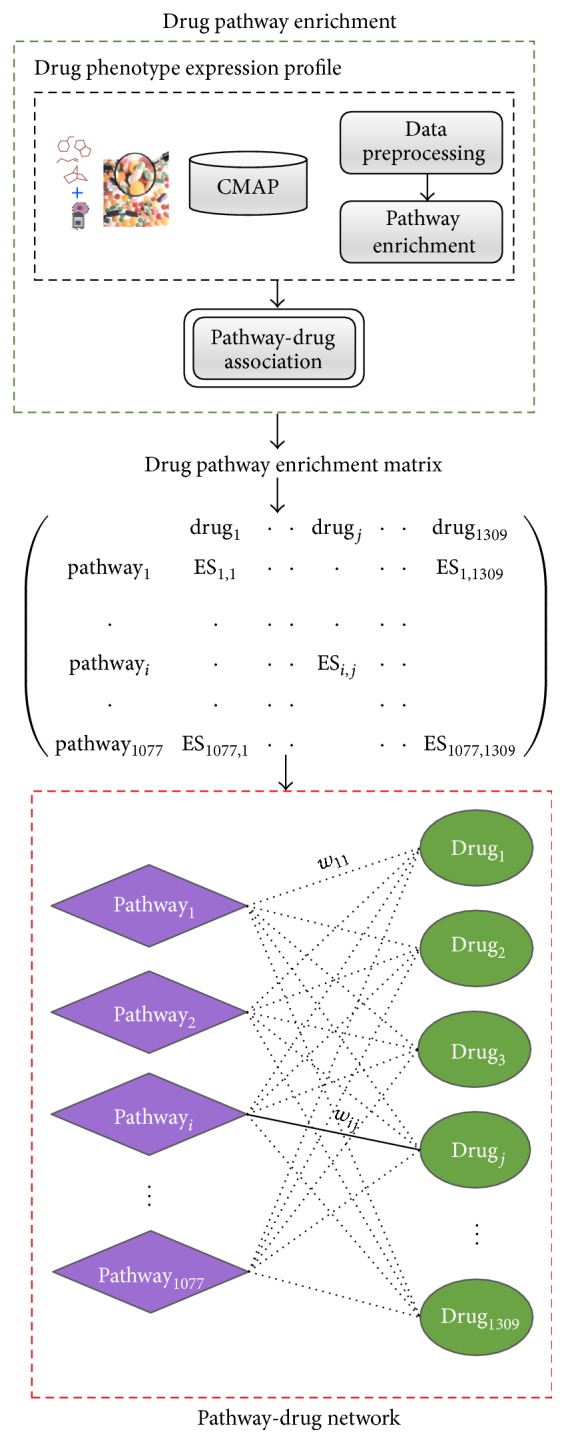
Drug pathway association and pathway-drug network. Associations between a drug and pathways are defined by drug pathway enrichment from drug phenotype expression profiles. The strength of ES_*i*,*j*_ represents the enrichment of pathway_*j*_ when treated with drug_*j*_.

To define a pathway-drug association, pathway-drug enrichment is established from the drug phenotype expression profile (CMap: Connectivity Map) [6, 7], which contains the gene expression profiles obtained from five different cancer cell lines treated with 1309 (v2) small drug molecules, most of which are FDA-approved drugs, for a total 6100 data points representing gene expression results with control vehicle samples. The CMap data are preprocessed, batch effects are removed, and pathway enrichments are estimated by GSEA as in previous studies [11, 26, 27]. As a result, each pathway (1077) has an ES for each drug molecule (1309). The strength of the ES indicates the association degree of a pathway with a drug. As shown in Figure 3, the pathway-drug association can be represented as a 1077 × 1309 matrix, where the columns list the drugs and the rows list the pathways.

### 2.3. Pathway-Drug Network Construction

A pathway-drug network was established from the drug pathway association profile. By using the pathway-drug enrichment matrix (Figure 3), the pathway-drug bipartite graph structure *G* = (*U*, *V*, *E*, *w*) was constructed, whose vertices can be divided into two disjoint sets: *U* (pathways) and *V* (drugs) such that every edge *e* ∈ *E* with weight *w* represents the enrichment of pathway *u*_*i*_ ∈ *U* by drug *v*_*j*_ ∈ *V*. In other words, each node in the network corresponds to a drug or pathway, and each edge corresponds to the association between them. It can be observed that drugs tend to bind with disease-specific pathways. All nodes were initially unlabeled as 0. Semisupervised learning on a network requires a small amount of labeled data with a large amount of unlabeled data.

**Figure 4:**
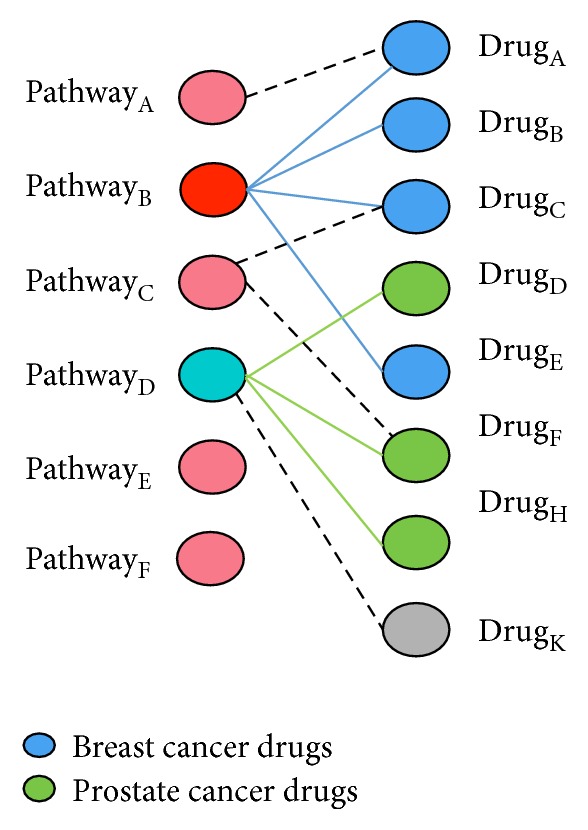
Similar drugs used for the same disease share most of the molecular pathway they target.

To use the constructed bipartite graph for drug repositioning, we made following assumption as in [4]: If pharmacologically different drugs induce the same phenotype of interest, then most of molecular pathways they target must be shared. In other words,* drugs used to treat the same disease (phenotype) target similar pathways*. For example, if we have some prior knowledge on certain drugs that are used to treat a specific disease, then most of the molecular pathway they target should be similar. In Figure 4, the blue drugs (breast cancer treatment drugs) target pathway “B,” and the green drugs (prostate cancer treatment drugs) target pathway “D.” From this information, it is can be concluded that drug “K” can likely be used to treat prostate cancer, when the weight (ES) is high enough. This is main assumption that we make in our proposed framework for pathway-based drug repositioning. Defining the initial knowledge (or initial labels for nodes) is also one of the key steps in this work.

### 2.4. Label Initialization on a Pathway-Drug Network

To initialize the pathway-drug labels for the *U (pathways)* and *V (drugs) *disjoint sets, we used disease-specific pathways inferred from the multiple gene expression profiles and known treatment drugs for the given phenotype (breast cancer) were obtained from three different public resources: the Maya Clinic, Cancer Organization, and TTD. The identified disease-specific pathways were mapped to the* U (pathways) set* and labeled as 1, and the remaining pathways were labeled as 0.

For the *V (drugs)* set, a more accurate prediction is possible if we can set the labels for the drug set in the pathway-drug network using previously known information about the disease-related drugs prior to using network propagation to predict drugs associated with the disease. Therefore, we first verified known drugs used for the treatment of the disease of interest using public drug-related sources, including the Maya Clinic database, Cancer Organization database, and TTD, and then determined the labels for the drug set in the pathway-drug network. These drugs were mapped to the *V (drugs)* set and labeled as 1, and the remaining drugs were labeled as 0.

### 2.5. Drug Repositioning by Semisupervised Learning

Once the initial labeling of the pathway-drug network was completed, we predicted the repositioned drugs by learning the drug nodes and pathway nodes with the network propagation algorithm. The bipartite graph can be defined as *G* = (*V*, *U*, *E*, *w*), where *V* and *U* are the node sets that are the disjoint node, in which the nodes of each node set are expressed as *v* and *u*, respectively. *E* is the set of edges between *V* and *U*, and *w* represents the weights of these edges. The weight of a specific edge is expressed as *w*(*v*, *u*). The function for the sum of all weight values for a node can be defined as *d*(*v*) = ∑_(*v*,*u*)_ *w*(*v*, *u*). Now, let us examine the network propagation algorithm based on the definition of the previously defined bipartite graph. First, the network propagation algorithm normalizes the weights of the bipartite graph using the following formula:

(1) $$\begin{array}{c} B=D_{v}^{-1/2}*W*D_{u}^{-1/2}. \end{array}$$

Here,* W* is a matrix containing the weights of the bipartite graph, *D*_*v*_ and *D*_*u*_ are the diagonal matrices with the values of *D*_*i*_*v*_*i*_*v*__ = *d*(*v*) and *D*_*i*_*u*_*i*_*u*__ = *d*(*u*), respectively, and *B* is the matrix of the normalized weights. Second, network propagation is performed for the bipartite graph using formulae (2) and (3), iterating over the objective function of the graph-based semisupervised learning algorithm.

For each *v* ∈ *V*,

(2) $$\begin{array}{c} f{\left(\;v\;\right)}^{t}=\left(\;1-\alpha \;\right)y\left(\;v\;\right)+\alpha \underset{u\in U}{\sum }B_{i_{v}i_{u}}f{\left(\;u\;\right)}^{t-1}. \end{array}$$

For each *u* ∈ *U*,

(3) $$\begin{array}{c} f{\left(\;u\;\right)}^{t}=\left(\;1-\alpha \;\right)y\left(\;u\;\right)+\alpha \underset{v\in V}{\sum }B_{i_{v}i_{u}}f{\left(\;v\;\right)}^{t-1}. \end{array}$$

Here, *t* is the number of iterations and *y* is the initial label of the corresponding node. The parameter *α* has a value between 0 and 1 and acts to regulate the relative weight of the initial label and the learned label. *y*(*v*) and *y*(*u*) are the initial labels for the drugs and pathway, respectively, whereas *f*(*v*) and *f*(*u*) are the final label scores. Finally, network propagation is completed when the values of *f*(*v*) and *f*(*u*) converge.

If the network propagation algorithm is executed over the pathway-drug bipartite according to the above method, the learned drugs label scores can be obtained. As the label score of a drug increases, the drug can be considered a more promising candidate for drug repositioning for the given phenotype. Therefore, we define the values of the final drug label scores as the drug repositioning scores and use them to predict disease-associated drugs from the pathway-drug network. In addition, all obtained label scores are normalized by the *Z*-score using the following equation:

(4) $$\begin{array}{c} Z_{{drug}_{i}}=\frac{l_{{drug}_{i}}-mean\left(l\right)}{std\left(l\right)}, \end{array}$$

where *l* is the label score vector for all drugs and *l*_drug_*i*__ is the final label score for drug_*i*_. For each drug, the corresponding *p* value was estimated based on the *Z*-score for Gaussian distribution. For more conservative results, we chose drugs with *p* < 0.001 as promising drug candidates for drug repositioning for the given disease. The selected promising drug candidates are evaluated by our validation methods and chosen for further investigation.

## 3. Results and Discussion

We tested our proposed framework to reposition 1309 drugs for breast cancer.

### 3.1. Finding Disease-Specific Pathways in Breast Cancer

To obtain breast cancer-specific pathways, we used publicly available breast cancer expression profiles (GSE15852 [28], GSE20437 [29], GSE2043 [30], and GSE2990 [31]) from the Gene Expression Omnibus (GEO) [32].  Table 1 shows the detailed characteristics of the expression profiles used in our study. Each dataset was preprocessed using RMA techniques [33] and implemented in R using the BioConductor package, which includes a large number of metadata packages appropriate for different types of microarrays. Supplementary Figure  1, in Supplementary Material available online at http://dx.doi.org/10.1155/2016/7147039, shows the results of preprocessing. For each dataset, the corresponding annotation databases were downloaded separately, and each probe was mapped to a HUGO [34] gene symbol; a probe was discarded if it did not match any symbol. In addition, if a gene had multiple probes (many-to-one), the gene expression values were averaged over the probes.

The human metabolic and signaling pathways were obtained from the Molecular Signature Database (MSigDB) [35]. As shown in Table 2, we chose the canonical pathways in the curated gene sets that contain 1077 pathways collected from KEGG [36], Reactome [37], and BioCarta (http://www.biocarta.com/).

For each dataset, a pathway was defined as breast cancer enriched by GSEA when *p* < 0.01. To integrate, the enriched pathways with nominal *p* values less than 0.01 (*p* < 0.01) were selected as significant pathways for each expression profile, and their union was defined as the “disease-specific pathways.” Table 3 shows the number of enriched pathways for each dataset and the integrated pathways obtained by taking their union.  Table 4 shows an example of enriched pathways in breast cancer by using experiment dataset (GSE2990). In the Supplementary Material, Tables 1 [Table tab2] [Table tab3]–4 provide the GSEA analysis results for each cancer expression profile and list the identified disease-specific pathways that were used for label initialization on the pathway-drug network.

### 3.2. Breast Cancer Drug Repositioning Using the Proposed Approach

From the four different breast cancer expression profiles, 143 pathways were identified as significantly enriched. On the pathway-drug network, these pathways were mapped to the *U (pathways) set and *initially labeled as 1, and the remaining 934 pathways were labeled as 0. In addition, known drugs used for the treatment for breast cancer were obtained from three different public resources, the Maya Clinic, Cancer Organization, and TTD. Sixty-one drugs approved to treat breast cancer were obtained from the Maya Clinic, 49 drugs were obtained from the Cancer Organization, and 11 drugs were obtained from TTD. Next, after mapping these drugs to the drug pathway network only 10 drugs were successfully mapped. Moreover, the 10 mapped drugs (tamoxifen, letrozole, doxorubicin, vinblastine, exemestane, aminoglutethimide, methotrexate, paclitaxel, megestrol, and fulvestrant) were labeled as 1 on* V (drugs)*, whereas all remaining drugs (1299) were labeled as 0.

**Figure 5:**
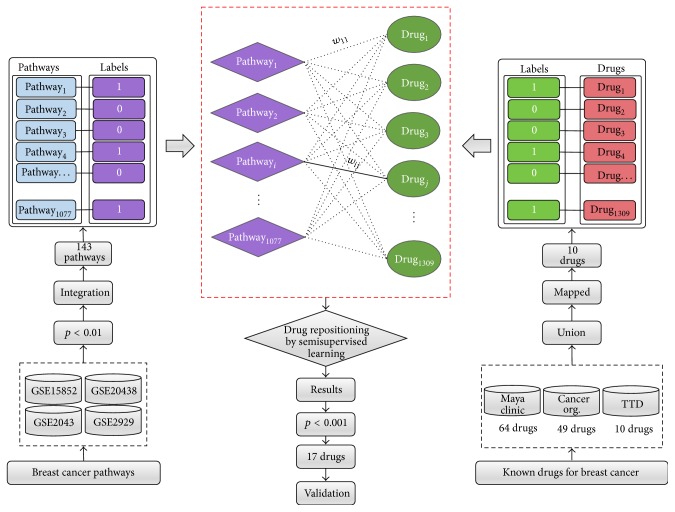
Breast cancer drug repositioning. Ten known drugs approved to treat breast cancer were obtained from the Maya Clinic, Cancer Org, and TTD. A total of 143 breast cancer-specific pathways were identified from multiple breast cancer expression profiles. Successfully mapped pathways and drugs were labeled as 1. Once labels were initialized on the pathway-drug network, we repositioned drugs for breast cancer using semisupervised learning. Predicted drugs with *p* < 0.001 were considered promising candidate drugs, and their associations with breast cancer were investigated using two different validation methods.

Once the initial labels of the pathway-drug network were chosen, we predicted promising candidates related to breast cancer using semisupervised network propagation, as shown in Figure 5. As a result, we considered 17 drugs with *p* < 0.001, as shown in Table 5, and found that 10 of them are already known drugs. The remaining seven drugs were considered as promising drug candidates for breast cancer and used for further validation to examine their association with breast cancer.

### 3.3. Validation of Promising Candidate Drugs

To validate the predicted drugs, we recommend the use of two different methods. Drugs that have been successfully validated by both methods are considered to be confirmed for repositioning for breast cancer.

#### 3.3.1. Biological Validation

Biological validation was performed by manually checking the evidence in the biological literature on promising drug candidates. We manually searched for any possible indication of the repositioned drugs for breast cancer. As shown in Table 6, for each promising drug candidate, several different lines of evidence in the literature were found indicating its possible use for breast cancer. Based on these results, we concluded that six drugs of seven drugs were confirmed by biological validation for their new usage in breast cancer treatment, with phenoxybenzamine not being confirmed.

#### 3.3.2. Computational Evaluation on the Validation Network

In drug repositioning, it is difficult to compare and evaluate the performances of computational methods. To address this issue, several recent studies have focused on curating a comprehensive and public catalog of existing drug indications using a manual process [4].

**Figure 6:**
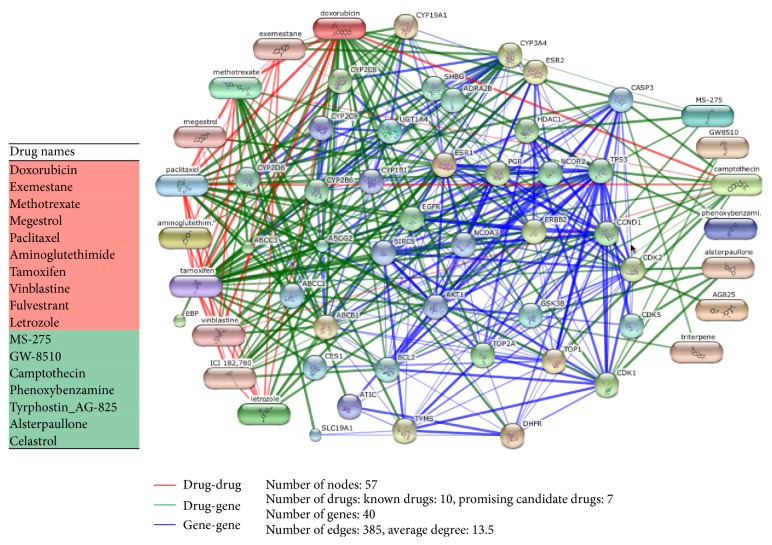
Known drugs and promising drug candidates on the validation network. The validation network for 16 drugs was constructed from STITCH. Each node is a drug or a gene. The green edges represent drug-gene interactions, and the red edges indicate drug-drug interactions; the blue edges represent gene-gene relationships obtained from STRING. Wider edges reflect stronger relationships between nodes. For easier implementation and visualization, a maximum of 40 neighbors of drugs (17 nodes) with a weight criterion of *r* > 0.4 were selected. As indicated in the figure, some drugs have significant topological features on the validation network.

Therefore, to develop a better evaluation method using computational methods, a validation network was constructed using information on three different relationships, drug-drug, drug-gene, and gene-gene, from the STITCH and STRING databases [38]. The drug-drug relationship information was obtained from the STITCH (v4) [39] database, which contains data on the interactions between small molecules and the edges between two chemicals that are expressed using a score between 0 and 900 defined from the chemical similarity between drugs. The drug-gene network was constructed from STITCH (for human) protein-chemical interactions with the help of the STRING database which provides 4,523,609 relationships for humans with the correlations between proteins and chemicals recorded as scores using information obtained from experimental results, text-mining, or predicted correlations. The gene-gene network was constructed from the STRING database, where A PPI network can be described as a complex system of proteins linked by interactions. Two proteins or genes that physically interact are represented as adjacent nodes connected by an edge. Each protein id (unipro id) is converted to the corresponding gene symbols using annotation databases provided in the STRING protein-protein interaction database. For computational evaluation, we have selected a maximum of 40 neighbors of drugs (17 drugs) with a weight criterion of *r* > 0.4 from the validation network derived from STITCH. The constructed validation network is illustrated in Figure 6.

To investigate the node properties in a network, network topology measurements (degree centrality and betweenness) and linkage analysis (PageRank) are often used. Degree centrality represents the number of interactions/edges/connections for a node. Biological networks are mostly scale-free networks, in which most nodes have few edges and a small number of nodes (hub) have a very high degree centrality. Betweenness is measured by the shortest paths between all nodes in the network and nodes that have the “shortest path” going through them are called bottlenecks. These hub and bottleneck nodes are topologically important and are usually functionally essential nodes (genes and drugs that have significant biological roles). Nodes connected to the hub and bottleneck node directly can also be functionally important. In addition, link analysis is a technique used to evaluate relationships (connection weights). The PageRank is a popular link analysis algorithm based on idea that a node should be significant if other significant nodes contain links to it.

By answering the following biological questions for the promising drug candidates, we identified the most promising drugs among them.

- Which candidate drug has an interesting/important relationship (connections) with known drugs?
- Which candidate drug has the hub/bottleneck property on the validation network?
- Which candidate drugs are connected to known breast cancer target genes?

For this purpose, we checked the network properties of promising drug candidates on the validation network using degree centrality, betweenness, and PageRank. Among them, the network topology measurements (degree centrality and betweenness) are designed to produce a ranking which allows indication of the most important vertices and not designed to measure the influence of neighbor nodes in general. Therefore, for better validation of promising candidates on validation network, PageRank algorithm seems to be more preferable which evaluates the nodes by considering their connection weights to the influential neighbors nodes.

**Figure 7:**
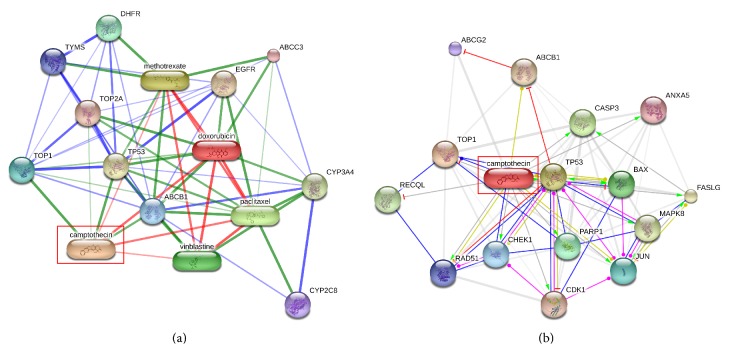
The candidate drug camptothecin on the validation network. (a) Camptothecin has a strong relationship (chemical similarity) with known breast cancer drugs: doxorubin, paclitaxel, vinblastine, and methotrexate. (b) Camptothecin has direct target relationship with the genes playing active roles in breast cancer including TOP1, ABCB1, TOP2A, CASP3, and TP53 (neighbors). Moreover, it has an indirect relationship with the breast cancer target gene EGFR.

From the results shown in Table 7, the popular breast cancer drug “tamoxifen” was identified as the most important hub node with degree centrality of 0.661 on the validation network. Among the promising drug candidates,* camptothecin* showed the hub node property with the highest degree centrality (0.232) among the other five (MS-275, GW-8510, phenoxybenzamine, tyrphostin_AG-825, and alsterpaullone).  Table 8 shows the neighbor nodes of the camptothecin on the validation network where it has a strong chemical similarity with the known drugs doxorubicin, paclitaxel, vinblastine, and methotrexate. A close look at this relationship is shown in Figure 7(a), and this evidence seems to point to the possibility of using the camptothecin for breast cancer treatment because structurally similar drugs usually bind the same disease targets. In addition, from Table 8 and Figures 7(a) and 7(b), it can be seen that camptothecin has a strong target relation with the genes that play active role in breast cancer including TOP1, ABCB1, TOP2A, CASP3, and TP53 (neighbors) and EGFR (second-degree neighbor). TOP1 and TOP2A were reported to inhibit the breast cancer resistant proteins [40]. ABCB1 is known as prognostic factor in breast cancer patients [41]. CASP3 expression loss represents an important cell survival mechanism in breast cancer patients [42] and it inhibits the growth of breast cancer cells. EGFR was one of the first identified important targets in breast cancer, and half of breast cancer cases overexpress EGFR.

**Figure 8:**
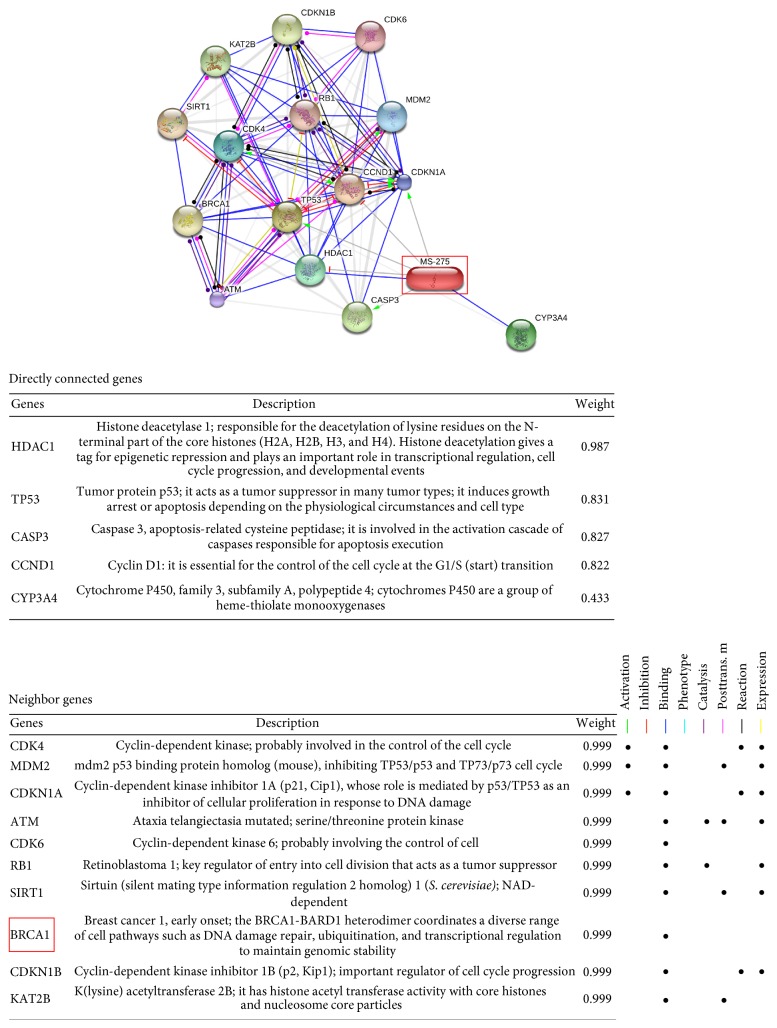
The candidate drug MS-275 on the validation network. MS-275 has a strong target relationship with the breast cancer genes HDAC1, TP53, CASP3, CCND1, and CYP3A4. Furthermore, it has an indirect relationship with the well-known breast cancer gene BRCA1.

The candidate drugs MS-257 and alsterpaullone showed relatively higher degree centrality values among the remaining drugs.  Table 9 and Figure 8 show the neighbor nodes relationship of MS-257 on the validation network, where it has strong target relationships with the genes HDAC1, TP53, CASP3, CCND1, and CYP3A4. Overexpression of HDCA1 represents clinicopathological indicators of disease progression in human breast cancer [43]. CCDN1 was reported to be a therapeutic target in breast cancer [44], and it has an indirect relationship with breast cancer susceptibility gene BRCA1. The betweenness results are summarized in Table 10. Among promising drug candidates only camptothecin and MS-275 showed some bottleneck node properties. Tamoxifen was defined as the most important bottleneck drug for breast cancer. Finally, we evaluated the connection weights of candidate drugs on the validation network using PageRank algorithm. We chose the alpha parameter as 0.85, which is the most commonly used value for this parameter with original Google PageRank algorithm. As shown in Table 11, camptothecin (0.257), alsterpaullone (0.102), and MS-275 (0,088) exhibited higher ranking scores than the other promising candidate drugs.

From the evidences shown above, we concluded that* camptothecin*,* MS-257*, and* alsterpaullone* exhibited the strongest network property evidences for breast cancer on the validation network. In general, all of the promising candidates successfully passed the computational evaluation on the network.

**Figure 9:**
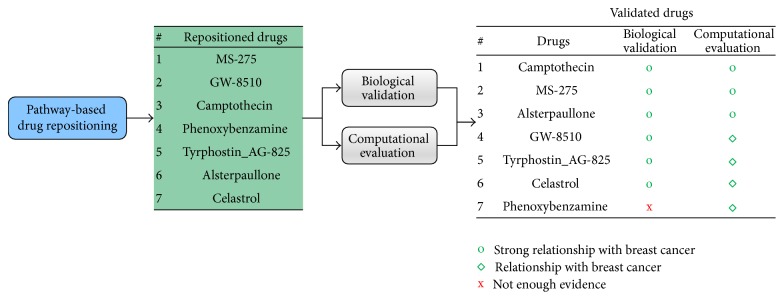
Validated drugs. Candidate drugs with successful results for both the biological validation and computational evaluation are considered repositioned drugs for breast cancer.

After performing biological and computational evaluations of the promising candidate drugs, we selected* camptothecin* as the most promising candidate because it was the most successful in both evaluation processes. For MS-278, GW-85, AG825, alsterpaullone, and celastrol, there was strong literature evidence with a reasonable network property. Thus, as shown in Figure 9, camptothecin, MS-278, alsterpaullone, GW-85, and AG825 and were validated as repositioned drugs and indicated for further investigation in breast cancer treatment.

## 4. Summary

We introduced a new systematic framework for disease-specific drug repositioning from integrated gene expression profiles on a pathway-drug network constructed from drug phenotype expression profiles (CMap) using semisupervised learning. The proposed pathway-based drug repositioning process showed encouraging results when using four different disease expression profiles to predict candidate drugs for disease-specific repositioning.

Two different methods were employed to evaluate the repositioned drugs. The drugs that passed both evaluation methods successfully were considered the most promising drugs to target breast cancer. As a result, several drugs, including camptothecin, MS-275, alsterpaullone, GW-8510, AG 825, and celastrol were identified as possible drugs to be repositioned to treat breast cancer, and these results are supported by multiple lines of evidence in the public literature. Specifically,* camptothecin* was the most promising drug candidate because it showed a high network property on the validation network and was supported by evidence in the literature.

Despite the interesting results, our method for drug repositioning was developed and validated in only using integrated mRNA gene expression profiles. However, the strategy can be easily improved to include other experimental data types, such as RNA-seq, miRNA, DNA-methylation, and single nucleotide polymorphism (SNP) information. Finally, the increasing number of genomic and pharmaceutical databases necessitates the further development of the method to identify new drugs and targets for rare cancer subtypes, develop personalized medicine, and design targeted cancer therapies.

## Supplementary Material

Supplementary file 1 contains Figures (1 and 2) of the preprocessing results and enrichment heatmaps for each datasets.
Supplementary files (2, 3, 4, and 5) contain enrichment analysis results of each datasets in tab-limited format.

## Figures and Tables

**Table 1:** Breast cancer gene expression datasets.

#	Dataset id	Samples (control/case)	Probes	Platform	References
1	GSE15852	86 (43/43)	22283	GPL96 (HG-U133A)	Pau Ni et al. [28]
2	GSE20438	42 (18/26)	22283	GPL96 (HG-U133A)	Graham et al. [29]
3	GSE2043	286 (180/106)	22283	GPL96 (HG-U133A)	Wang et al. [30]
4	GSE2929	193 (64/129)	22283	GPL96 (HG-U133A)	Sotiriou et al. [31]

The gene expression datasets were downloaded from the NCBI Gene Expression Omnibus (GEO).

**Table 2 tab2:** Pathway data.

Database	Pathways	# of gene sets	URL
MSigDB (c2-canonical pathways)	KEGG	186	http://www.genome.jp/kegg/
	Reactome	674	http://www.reactome.org/
	BioCarta	217	http://www.biocarta.com/

Total		1077	

**Table 3 tab3:** Breast cancer disease-specific pathways for each dataset.

	GSE15852	GSE20438	GSE2043	GSE2929	Integrated pathways
# of pathways *p* < 0.01	109	7	17	21	**143**

Enriched pathways were identified by GSEA. For integration, the pathways with (*p* value < 0.01) were selected as significant pathways for each expression profile and their union was defined as “disease-specific pathways.”

**Table 4:** Breast cancer pathways from GSE2990 (*p* < 0.01).

#	Name	ES	NES	*p* value
1	REACTOME_DEFENSINS	−0.767	−1.599	0.008230452
2	REACTOME_ORGANIC_CATION_ANION_ZWITTERION_TRANSPORT	0.771	1.668	0.004016064
3	REACTOME_G0_AND_EARLY_G1	−0.771	−1.601	0.008213553
4	REACTOME_CELL_SURFACE_INTERACTIONS_AT_THE_VASCULAR_WALL	−0.601	−1.670	0
5	REACTOME_SYNTHESIS_OF_BILE_ACIDS_AND_BILE_SALTS_VIA_7ALPHA_HYDROXYCHOLESTEROL	0.756	1.644	0.00617284
6	REACTOME_PECAM1_INTERACTIONS	−0.855	−1.685	0.001972387
7	REACTOME_GABA_SYNTHESIS_RELEASE_REUPTAKE_AND_DEGRADATION	0.773	1.558	0.003891051
8	REACTOME_ACTIVATION_OF_THE_PRE_REPLICATIVE_COMPLEX	−0.773	−1.613	0.008438818
9	BIOCARTA_GATA3_PATHWAY	0.746	1.688	0.002004008
10	REACTOME_NEUROTRANSMITTER_RELEASE_CYCLE	0.709	1.747	0
11	BIOCARTA_G2_PATHWAY	−0.705	−1.704	0.00625
12	REACTOME_PASSIVE_TRANSPORT_BY_AQUAPORINS	−0.757	−1.718	0.001865672
13	REACTOME_PYRIMIDINE_METABOLISM	−0.702	−1.726	0.001964637
14	BIOCARTA_ACTINY_PATHWAY	−0.734	−1.749	0.001945525
15	REACTOME_SYNTHESIS_OF_GLYCOSYLPHOSPHATIDYLINOSITOL_GPI	0.703	1.699	0.001976285
16	REACTOME_ENDOGENOUS_STEROLS	−0.762	−1.694	0.005703422
17	REACTOME_GLYCOSPHINGOLIPID_METABOLISM	0.590	1.763	0.006048387

Supplementary files 2, 3, 4, and 5 provide the full pathway enrichment analysis results for the breast cancer expression profiles.

**Table 5:** Predicted drugs after pathway-based drug repositioning.

Drug name	Ranking score	*Z*-score	*p* value	Description
Doxorubicin^*∗*^	0.999	8.935	0	It works by intercalating DNA, with the most serious adverse effect being life threatening heart damage.
Exemestane^*∗*^	0.803	6.880	2.99*E* − 12	Tyrosine kinase inhibitor which selectively inhibits HER2
Methotrexate^*∗*^	0.708	5.892	1.91*E* − 09	blocks the production of steroids derived from cholesterol and is clinically used in the treatment of Cushing's syndrome and metastatic breast cancer.
Megestrol^*∗*^	0.668	5.464	2.33*E* − 08	It binds to and inhibits the enzyme dihydrofolate reductase, resulting in inhibition of purine nucleotide and thymidylate synthesis and, subsequently, inhibition of DNA and RNA syntheses.
Paclitaxel^*∗*^	0.646	5.235	8.26*E* − 08	It binds to and stabilizes microtubules, preventing their depolymerization and so inhibiting cellular motility, mitosis, and replication.
Aminoglutethimide^*∗*^	0.637	5.148	1.32*E* − 07	It blocks the production of steroids derived from cholesterol and is clinically used in the treatment of Cushing's syndrome and metastatic breast cancer.
Tamoxifen^*∗*^	0.634	5.113	1.59*E* − 07	It is an antagonist of the estrogen receptor in breast tissue via its active metabolite, hydroxytamoxifen. In other tissues such as the endometrium, it behaves as an agonist and thus may be characterized as a mixed agonist/antagonist.
Vinblastine^*∗*^	0.625	5.020	2.59*E* − 07	It is an antimicrotubule drug used to treat certain kinds of cancer, including Hodgkin's lymphoma, non-small cell lung cancer, breast cancer, head and neck cancer, and testicular cancer.
Fulvestrant^*∗*^	0.604	4.802	7.86*E* − 07	It is drug treatment of hormone receptor-positive metastatic breast cancer in postmenopausal women with disease progression following antiestrogen therapy. It is an estrogen receptor antagonist with no agonist effects, which works by downregulating the estrogen receptor.
Letrozole^*∗*^	0.579	4.539	2.83*E* − 06	It is an oral nonsteroidal aromatase inhibitor for the treatment of hormonally responsive breast cancer.
MS-275^*∗∗*^	0.530	4.023	2.87*E* − 05	Entinostat, also known as SNDX-275 and MS-275, is a benzamide histone deacetylase inhibitor undergoing *clinical trials* for treatment of various cancers.
GW-8510^*∗∗*^	0.477	3.467	0.000263332	Cyclin-dependent kinase 5 inhibitors: inhibition of dopamine transporter activity.
Camptothecin^*∗∗*^	0.475	3.452	0.000278495	It is an alkaloid isolated from the stem wood of the Chinese tree, *Camptotheca acuminata*. This compound selectively inhibits the nuclear enzyme DNA topoisomerase.
Phenoxybenzamine^*∗∗*^	0.461	3.303	0.000478379	It is an alpha-adrenergic antagonist with long duration of action. It has been used to treat hypertension and as a peripheral vasodilator.
Tyrphostin_AG-825^*∗∗*^	0.447	3.159	0.000792504	It is tyrosine kinase inhibitor, which selectively inhibits HER2.
Alsterpaullone^*∗∗*^	0.447	3.150	0.000815292	CDC2 protein kinase, antiangiogenic potential of small molecular inhibitors of cyclin-dependent kinases in vitro.
Celastrol^*∗∗*^	0.442	3.100	0.000966191	Celastrol is a remedial ingredient isolated from the root extracts of “*Tripterygium wilfordii*” (Thunder of God vine) and “*Celastrus regelii*.” In “in vitro” and “in vivo” animal experiments, celastrol exhibits antioxidant, anti-inflammatory, anticancer, and insecticidal activities.

^*∗*^ Known breast cancer drug. ^*∗∗*^Potential drug candidate for repositioning.

**Table 6:** Literature evidences for the promising drug candidates for breast cancer.

Promising drugs	Biological validation	Related literature for possible usage for breast cancer
MS-275^*∗∗*^	**o**	(i) [45]: the potential anticancer activity of MS-275 in combination with pentoxifylline in panel of cell lines and human breast cancer xenograft model in vitro and in vivo. (ii) [46]: MS-275 sensitizes TRAIL-resistant breast cancer cells, inhibits angiogenesis and metastasis, and reverses epithelial-mesenchymal transition in vivo. (iii) [47]: HDAC inhibitors (MS-275) enhance the apoptosis-inducing potential of TRAIL in breast carcinoma.

GW-8510^*∗∗*^	⋄	(i) [48]: repositioning of a cyclin-dependent kinase inhibitor GW-8510 as a ribonucleotide reductase M2 inhibitor to treat human colorectal cancer. In addition, GW-8510 induced autophagic cell death. (ii) [49]: in cell viability tests, four candidate drugs, GW-8510, etacrynic acid, ginkgolide A, and 6-azathymine, are identified as having high inhibitory activities against cancer cells.

Camptothecin^*∗∗*^	**o**	(i) [50]: the camptothecin targets WRN protein: mechanism and relevance in clinical breast cancer. (ii) [51]: CRLX101, an investigational camptothecin-containing nanoparticle-drug conjugate, targets cancer stem cells and impedes resistance to antiangiogenic therapy in mouse models of breast cancer. (iii) [52]: STI571 sensitizes breast cancer cells to 5-fluorouracil, cisplatin, and camptothecin in a cell type-specific manner. (iv) [53]: acquired camptothecin resistance of human breast cancer MCF-7/C4 cells with normal topoisomerase I and elevated DNA repair. (v) known as “Happy Tree” in Chinese traditional cancer treatment.

Phenoxybenzamine^*∗∗*^	**x**	(i) Not enough evidence.

Tyrphostin_AG-825^*∗∗*^	**o**	(i) [54]: C-Src activation by ErbB2 leads to attachment-independent growth of human breast epithelial cells. (ii) [55]: using in vivo mouse models of breast cancer: using gefitinib, ERBB1 inhibition rapidly inhibits tumor cell motility and invasion but not intravasation, whereas ERBB2 inhibition by AG825 rapidly blocks intravasation. (iii) [56]: tyrphostin AG 825 has been used in combination with hypericin-mediated photodynamic therapy (HY-PDT) for evaluating its therapeutic effects in HER2 overexpressing human breast cancer cells.

Alsterpaullone^*∗∗*^	**o**	(i) [57]: the antitumor effects of ALP through induction of apoptosis in breast cancer and leukemia cells. Identification of alsterpaullone as a novel small molecule inhibitor to target group 3 medulloblastoma. (ii) [58]: baicalein blocked survivin expression in lung and breast cancer cells. Alsterpaullone is a CDC2 kinase inhibitor (43). Both CDC25 phosphatase and CDC2 kinase inhibitors enhanced the baicalein-induced cancer cell death.

Celastrol^*∗∗*^	**o**	(i) [59]: anticancer effect of celastrol on human triple negative breast cancer: possible involvement of oxidative stress, mitochondrial dysfunction, apoptosis, and PI3K/Akt pathways. (ii) [60]: celastrol induces that apoptosis of breast cancer cells and inhibits invasion via downregulation of MMP-9.

^*∗∗*^Potential drug candidate for repositioning.

**Table 7:** Degree centrality of promising drug candidates on the validation network.

Rank	Drug name	Degree centrality
1	Tamoxifen^*∗*^	0.661
2	Doxorubicin^*∗*^	0.554
3	Paclitaxel^*∗*^	0.536
4	Fulvestrant^*∗*^	0.268
5	Methotrexate^*∗*^	0.268
6	Camptothecin^*∗∗*^	0.232
7	Letrozole^*∗*^	0.214
8	Vinblastine^*∗*^	0.196
9	Exemestane^*∗*^	0.179
10	Megestrol^*∗*^	0.125
11	Aminoglutethimide^*∗*^	0.107
12	MS-275^*∗∗*^	0.089
13	Alsterpaullone^*∗∗*^	0.071
14	GW-8510^*∗∗*^	0.036
15	Phenoxybenzamine^*∗∗*^	0.036
16	Celastrol^*∗∗*^	0.036
17	Tyrphostin_AG-825^*∗∗*^	0.018

^*∗*^Known breast cancer drug. ^*∗∗*^Potential drug candidate for repositioning.

**Table 8:** The neighbors of candidate drug “camptothecin” on the validation network.

Nodes	Description	Weight
TOP1	Topoisomerase (DNA) I: the reaction catalyzed by topoisomerases leads to the conversion of one topological isomer of DNA to another.	0.999
CASP3	Caspase 3: apoptosis-related cysteine peptidase: it is involved in the activation cascade of caspases responsible for apoptosis execution.	0.965
TP53	Tumor protein p53: it acts as a tumor suppressor in many tumor types and induces growth arrest or apoptosis depending on the physiological circumstances and cell type.	0.965
Doxorubicin^**∗**^	It is a drug used in cancer chemotherapy; it works by intercalating DNA, with the most serious adverse effect being life threatening heart damage.	0.890
ABCG2	ATP-binding cassette, subfamily G (WHITE), member 2; xenobiotic transporter that may play an important role in the exclusion of xenobiotics from the brain. It may be involved in brain-to-blood efflux. It appears to play a major role in the multidrug resistance phenotype of several cancer cell lines.	0.873
CDK1	Cyclin-dependent kinase 1: it plays a key role in the control of the eukaryotic cell cycle. It is required in higher cells for entry into S phase and mitosis. p34 is a component of the kinase complex that phosphorylates the repetitive C-terminus of RNA polymerase II.	0.846
ABCB1	ATP-binding cassette, subfamily B (MDR/TAP), member 1; energy-dependent efflux pump responsible for decreased drug accumulation in multidrug-resistant cells.	0.843
BCL2	B-cell CLL/lymphoma 2: it suppresses apoptosis in a variety of cell systems including factor-dependent lymphohematopoietic and neural cells. It regulates cell death by controlling the mitochondrial membrane permeability. It appears to function in a feedback loop system with caspases. It inhibits caspase activity either by preventing the release of cytochrome c from the mitochondria and/or by binding to the apoptosis-activating factor (APAF-1).	0.820
Paclitaxel^**∗**^	It binds to and inhibits the enzyme dihydrofolate reductase, resulting in inhibition of purine nucleotide and thymidylate synthesis and, subsequently, inhibition of DNA and RNA syntheses.	0.812
CDK2	Cyclin-dependent kinase 2; involved in the control of the cell cycle; interacting with cyclins A, B1, B3, D, or E. Activity of CDK2 is maximal during S phase and G2.	0.754
Vinblastine^**∗**^	An antimicrotubule drug used to treat certain kinds of cancer, including Hodgkin's lymphoma, non-small cell lung cancer, breast cancer, head and neck cancer, and testicular cancer.	0.560
Methotrexate^**∗**^	It blocks the production of steroids derived from cholesterol and is clinically used in the treatment of Cushing's syndrome and metastatic breast cancer.	0.554
TOP2A	Topoisomerase (DNA) II alpha 170 kDa; control of topological states of DNA by transient breakage and subsequent rejoining of DNA strands.	0.431

^*∗*^Known breast cancer drug.

**Table 9:** The neighbors of candidate drug “MS-257” on the validation network.

Genes	Description	Weight
HDAC1	Histone deacetylase 1; responsible for the deacetylation of lysine residues on the N-terminal part of the core histones (H2A, H2B, H3, and H4). Histone deacetylation gives a tag for epigenetic repression and plays an important role in transcriptional regulation, cell cycle progression, and developmental events.	0.987
TP53	Tumor protein p53: it acts as a tumor suppressor in many tumor types and induces growth arrest or apoptosis depending on the physiological circumstances and cell type.	0.831
CASP3	Caspase 3, apoptosis-related cysteine peptidase; involved in the activation cascade of caspases responsible for apoptosis execution.	0.827
CCND1	cyclin D1; essential for the control of the cell cycle at the G1/S (start) transition.	0.822
CYP3A4	Cytochrome P450, family 3, subfamily A, polypeptide 4; cytochromes P450 are a group of heme-thiolate monooxygenases.	0.433

**Table 10:** Betweenness of promising drug candidates on the validation network.

Rank	Drug name	Betweenness
1	Tamoxifen^*∗*^	172
2	Paclitaxel^*∗*^	37
3	Doxorubicin^*∗*^	32
4	Camptothecin^*∗∗*^	13
5	Exemestane^*∗*^	13
6	Fulvestrant^*∗*^	12
7	Methotrexate^*∗*^	10
8	Vinblastine^*∗*^	6
9	Megestrol^*∗*^	6
10	Aminoglutethimide^*∗*^	2
11	Letrozole^*∗*^	2
12	MS-275^*∗∗*^	2
13	GW-8510^*∗∗*^	0
14	Phenoxybenzamine^*∗∗*^	0
15	Tyrphostin_AG-825^*∗∗*^	0
16	Alsterpaullone^*∗∗*^	0
17	Celastrol^*∗∗*^	0

^*∗*^Known breast cancer drug. ^*∗∗*^Potential drug candidate for repositioning.

**Table 11:** PageRank of promising drug candidates on the validation network (*α* = 0.85).

Rank	Drug name	Ranking score
1	Tamoxifen^*∗*^	0.990
2	Doxorubicin^*∗*^	0.692
3	Paclitaxel^*∗*^	0.663
4	Methotrexate^*∗*^	0.373
5	Fulvestrant^*∗*^	0.316
6	Camptothecin^*∗∗*^	0.257
7	Letrozole^*∗*^	0.252
8	Vinblastine^*∗*^	0.235
9	Exemestane^*∗*^	0.176
10	Aminoglutethimide^*∗*^	0.108
11	Alsterpaullone^*∗∗*^	0.102
12	Megestrol^*∗*^	0.101
13	MS-275^*∗∗*^	0.088
14	Phenoxybenzamine^*∗∗*^	0.080
15	GW-8510^*∗∗*^	0.026
16	Celastrol^*∗∗*^	0.023
17	Tyrphostin_AG-825^*∗∗*^	0.010

^*∗*^Known breast cancer drug. ^*∗∗*^Potential drug candidate for repositioning.
