# Supplementary material for: A Systematic Framework for Drug Repositioning from Integrated Omics and Drug Phenotype Profiles Using Pathway-Drug Network
Source: Biomed Res Int. 2016 Dec 26;2016:7147039. doi: 10.1155/2016/7147039 (PMC5233404; doi:10.1155/2016/7147039)
Supplement: Supplementary file 1 — contains Figures (1 and 2) of the preprocessing results and enrichment heatmaps for each datasets. Supplementary files (2, 3, 4, and 5) contain enrichment analysis results of each datasets in tab-limited format. [file 7147039.f1.docx]

Supplementary Materials 1


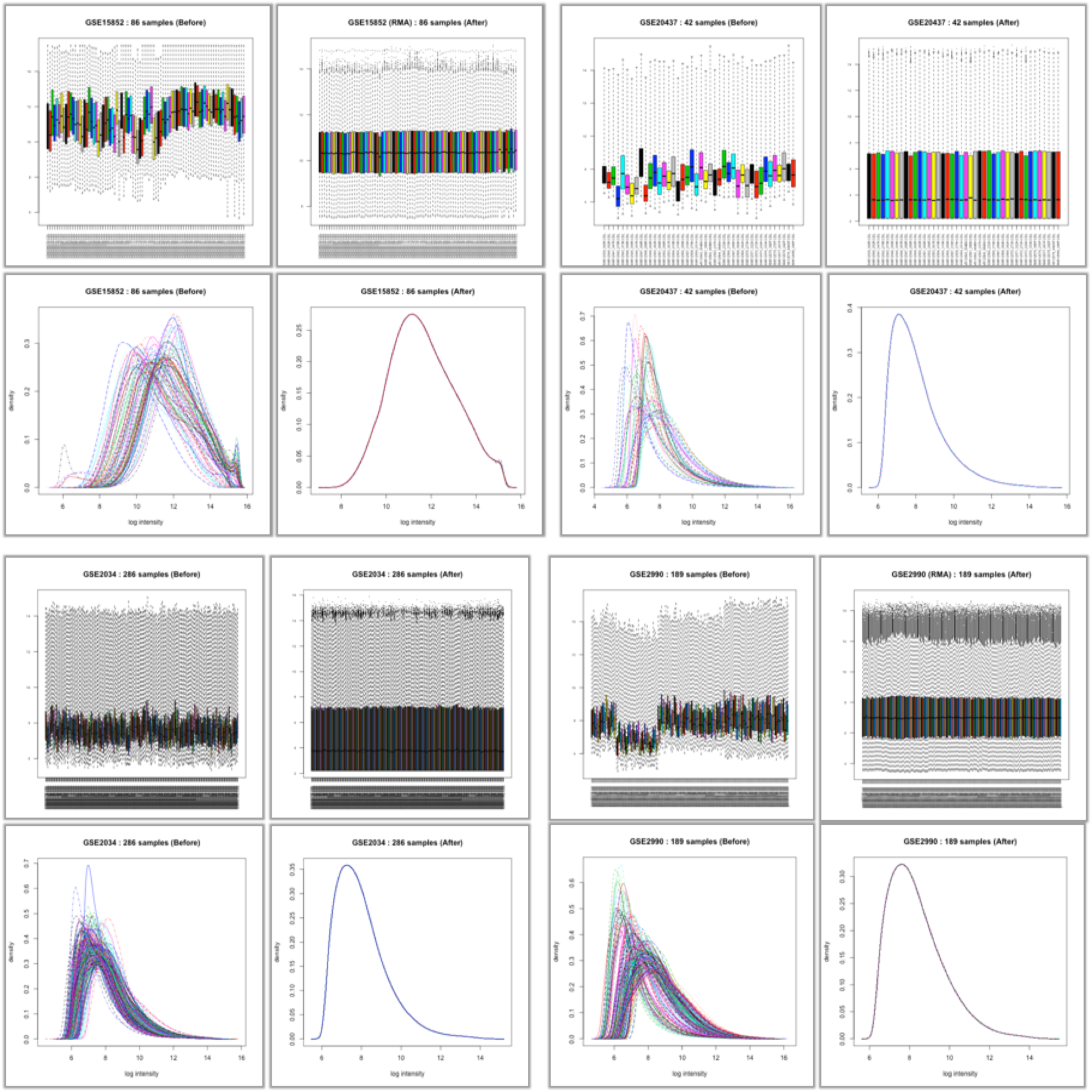


Figure 1: Preprocessing results of gene expression profiles.


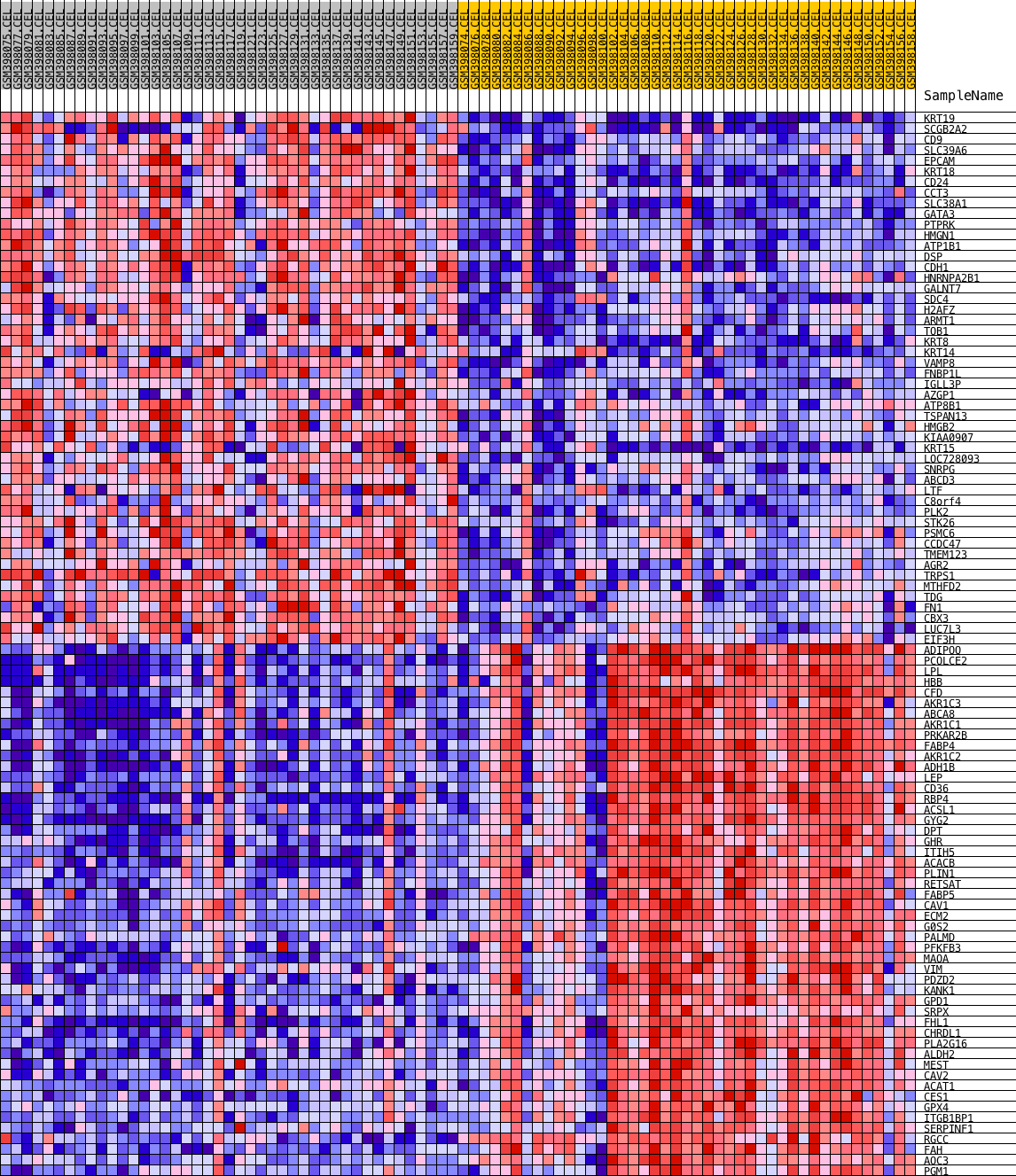


Figure 2:Enrichment heatmap of GSE15852.


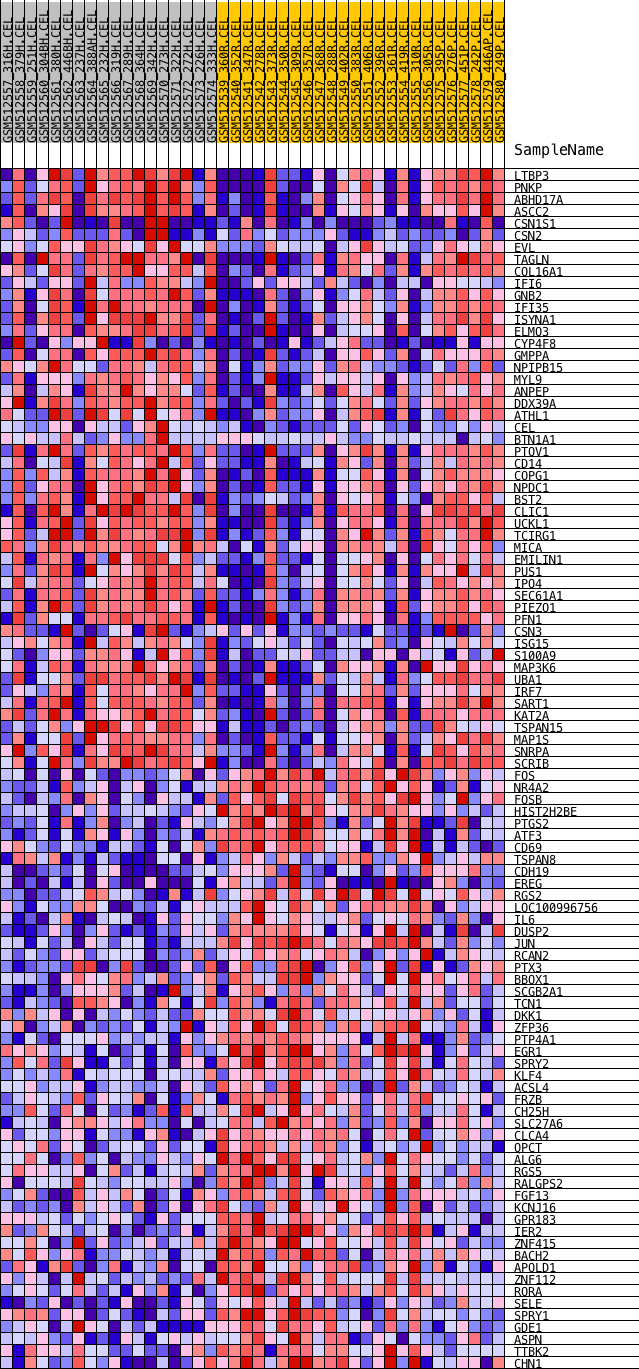


Figure 3: Enrichment heatmap of GSE20438.


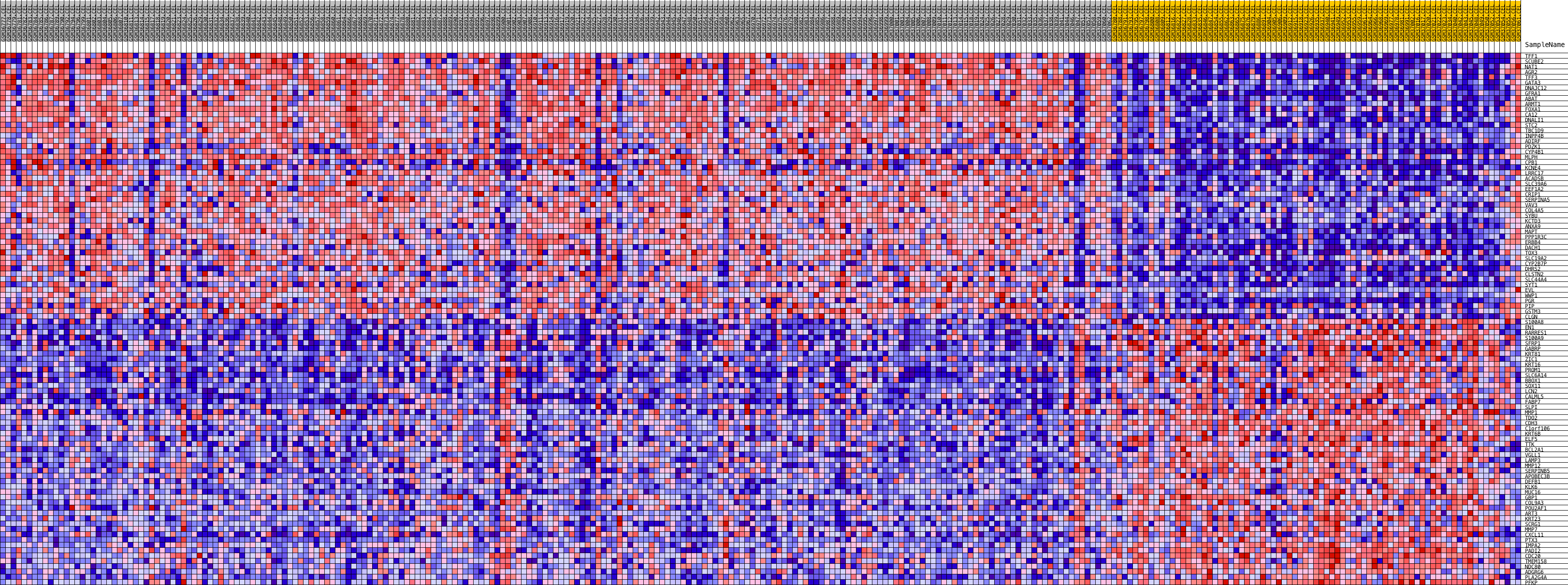


Figure 4: Enrichment heatmap of GSE2043.


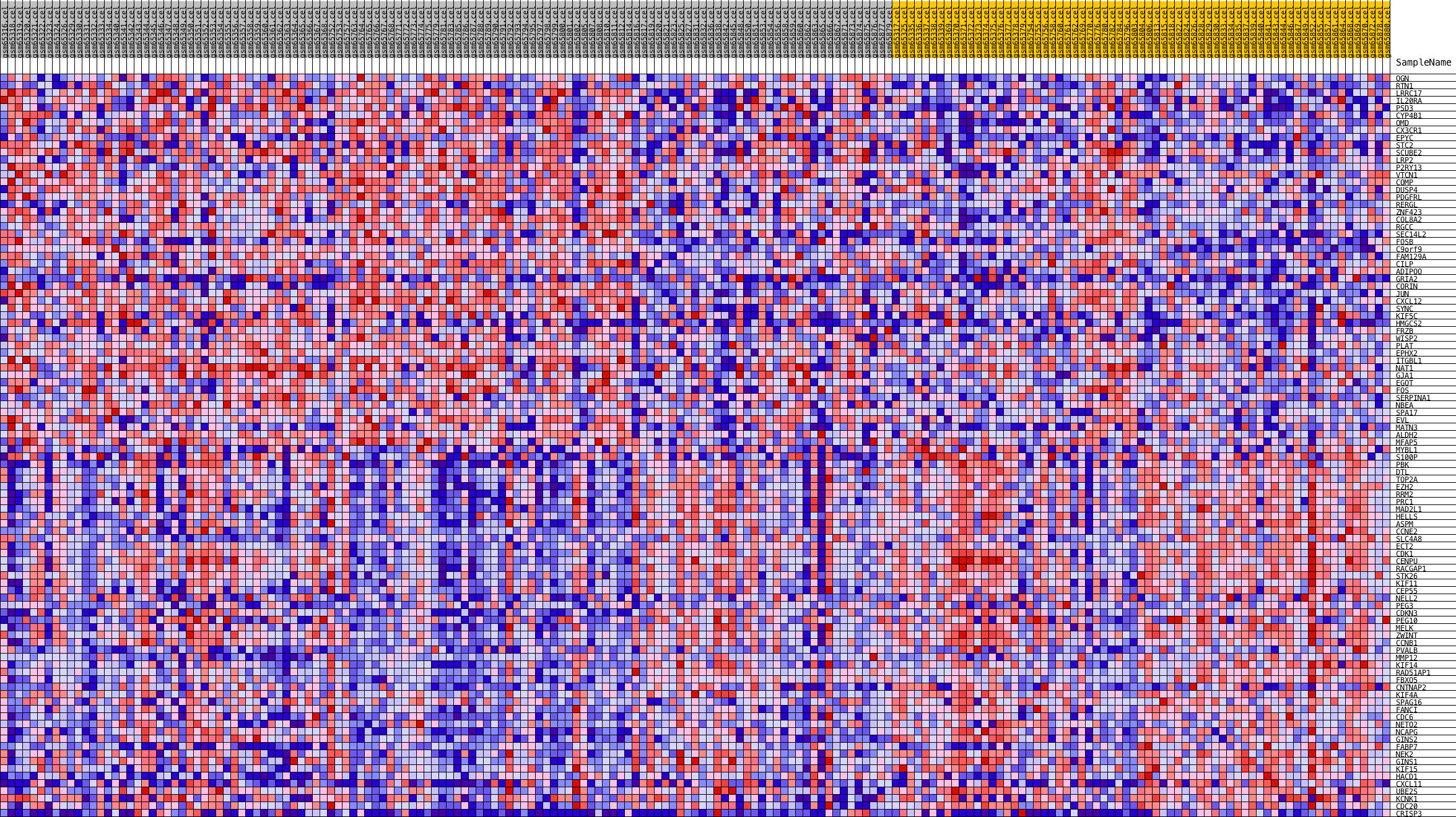


Figure 5: Enrichment heatmap of GSE2929.
